# Synthesis, crystal structure and Hirshfeld surface analysis of 2-[(2,4-di­methyl­benz­yl)sulfan­yl]pyrimidine-4,6-di­amine

**DOI:** 10.1107/S2056989025002440

**Published:** 2025-03-25

**Authors:** Gulrukh Salieva, Tursunali Kholikov, Rasul Ya Okmanov, Alimjon Matchanov, Khamid U Khodjaniyazov, Shakhnoza Kadirova, Batirbay Torambetov

**Affiliations:** a National University of Uzbekistan named after Mirzo Ulugbek 4 University St Tashkent 100174 Uzbekistan; b Tashkent Medical Academy, 2 Farabi St, Tashkent, 100109, Uzbekistan; c S. Yunusov Institute of the Chemistry of Plant Substances Academy of Sciences of Uzbekistan Mirzo Ulugbek St 77 Tashkent 100170 Uzbekistan; d Institute of Bioorganic Chemistry, Academy of Sciences of Uzbekistan, M. Ulugbek St, 83, Tashkent, 100125, Uzbekistan; Universidade de Sâo Paulo, Brazil

**Keywords:** crystal structure, mol­ecular structure, di­amino­pyrimidine-thiol, 2,4-di­methyl­benz­yl, Hirshfeld surface analysis

## Abstract

The title compound, C_13_H_16_N_4_S (DAMP-DMB), was synthesized through the reaction of 2,4-di­methyl­benzyl chloride with di­amino­pyrimidine-thiol. Single-crystal X-ray diffraction analysis confirmed that the compound crystallizes in the monoclinic crystal system, space group *P*2_1_/*c*. The asymmetric unit contains a single mol­ecular entity. Structural examination revealed the presence of a dimeric arrangement consolidated by N—H⋯N hydrogen-bonding inter­actions. Additionally, Hirshfeld surface analysis indicated that H⋯H, N⋯H, C⋯H, and S⋯H contacts account for 98.9% of the total inter­molecular inter­actions to the Hirshfeld surface.

## Abstract 2

The mol­ecular and crystal structures of 2-[(2,4-di­methyl­benz­yl)thio]­pyrimidine-4,6-di­amine were studied and Hirshfeld surfaces and fingerprint plots were generated to investigate the various inter­molecular inter­actions.

## 1. Chemical context

Di­amino-substituted pyrimidines are pyrimidine derivatives with important applications in pharmaceuticals and organic synthesis (Tolba *et al.*, 2022 ▸; Rosowsky *et al.*, 2004 ▸). These compounds play a crucial role in medicinal chemistry, in particular because of their anti­viral (Hocková *et al.*, 2004 ▸), anti­bacterial (Kandeel *et al.*, 1994 ▸), anti­malarial (Neekhara *et al.*, 2006 ▸) and anti­microbial activities (Holla *et al.*, 2006 ▸). Similarly, a 4,6-di­amino­pyrimidine-based derivative has showed potential anti­viral activity against dengue by targeting the NS2B/NS3 protease (Subasri *et al.*, 2017 ▸). Some organometallic complexes of di­amino­pyrimidine-thiol with tin and ruthenium exhibit anti­cancer activity (Grześkiewicz *et al.*, 2017 ▸; Silva *et al.*, 2020 ▸). Herein we report the crystal structure and Hirshfeld surface analysis of a newly synthesized organic compound, namely 2-[(2,4-di­methyl­benz­yl)sulfan­yl]pyrimidine-4,6-di­amine (DAMP-DMB).
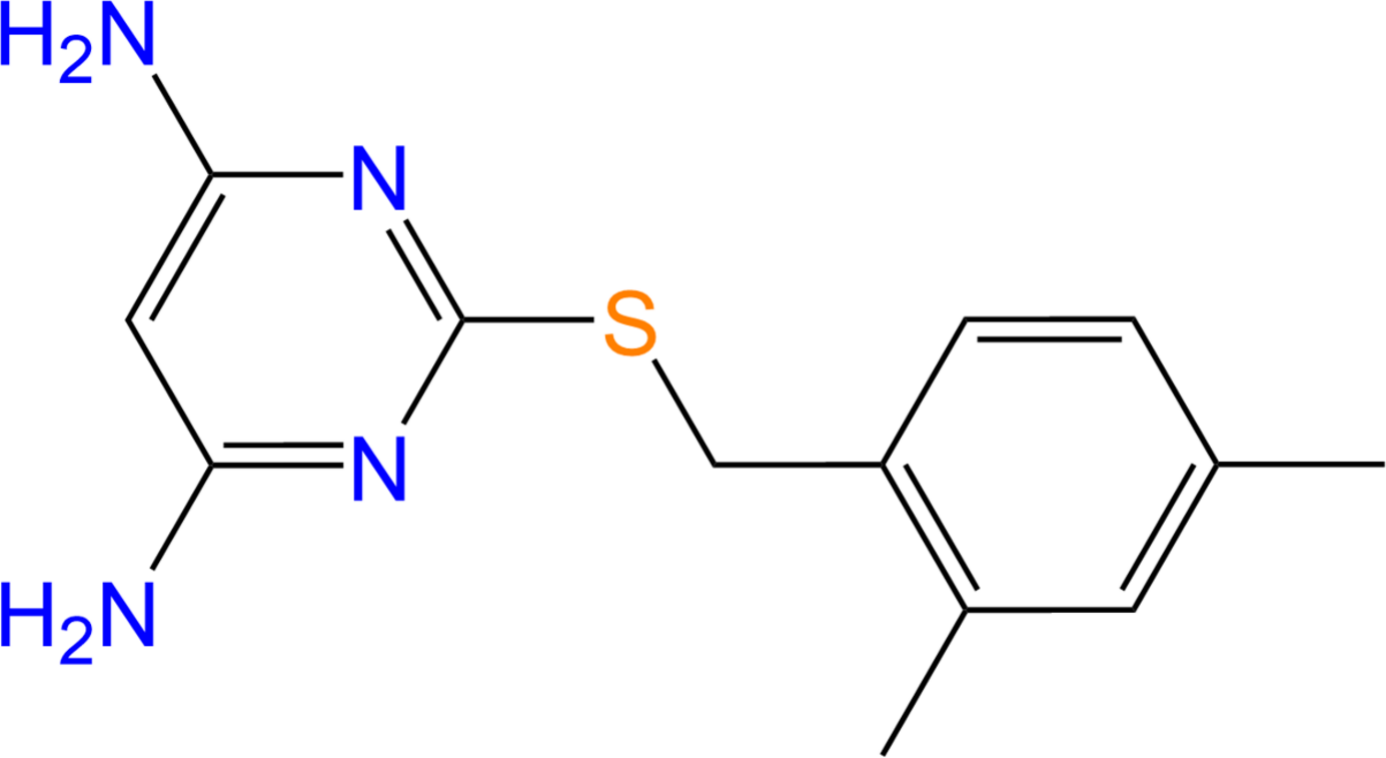


## 2. Structural commentary

**Figure 1:**
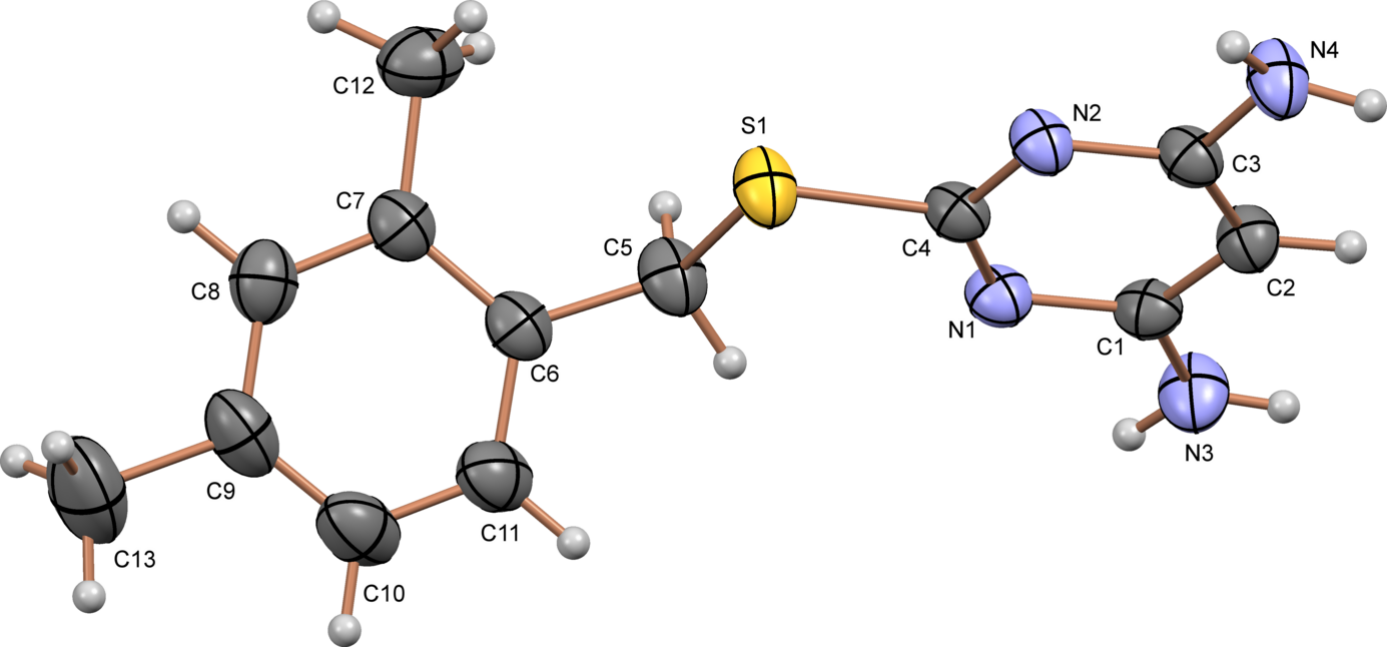
The mol­ecular structure of DAMP-DMB, with atomic displacement ellipsoids drawn at the 30% probability level, showing the atom labeling. Hydrogen atoms are represented as small spheres with arbitrary radii.

DAMP-DMB (Fig. 1 ▸) crystallizes in the monoclinic crystal system, space group *P*2_1_/*c* (14), with a single mol­ecule in the asymmetric unit. The amine groups on the pyrimidine ring are co-planar and the dihedral angle between the pyrimidine and phenyl rings is 63.03 (14)°. The torsion angles for the groups are N1—C4—S1—C5 = −6.7 (3)° and C11—C6—C5—S1 = −104.2 (3)° respectively. DAMP-DMB contains several hydrogen-bond donor and acceptor groups. However, due to the twisted conformation of the di­amino­pyrimidine group, the mol­ecule does not exhibit any intra­molecular hydrogen-bonding or π-stacking inter­actions.

## 3. Supra­molecular features

**Figure 2:**
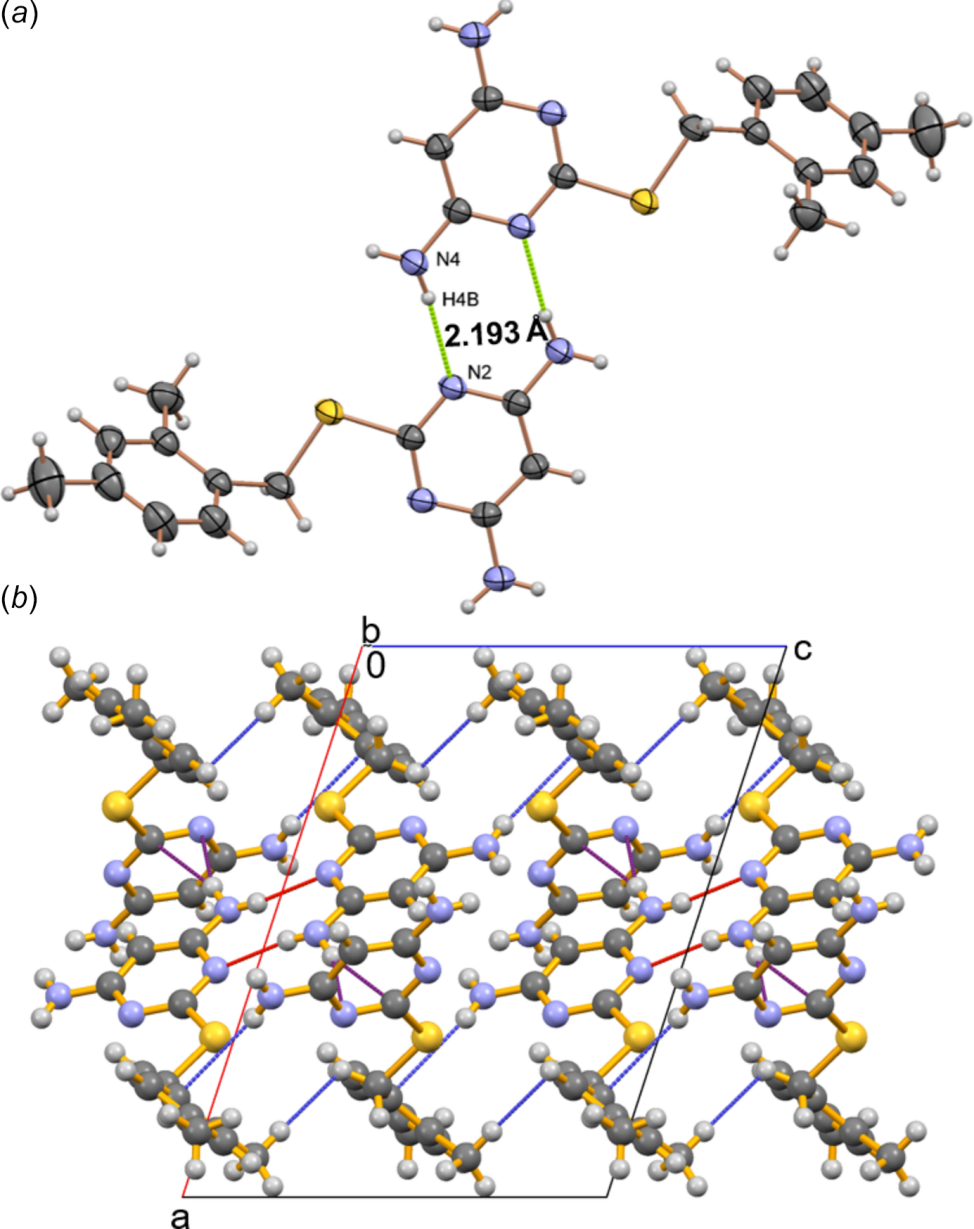
(*a*) The association between the mol­ecules of DAMP-DMB to form a dimer involving N4—H4*B*⋯N2 inter­actions and (*b*) view of the packing of mol­ecules and association of dimeric units along the *c* axis in the crystal structure of DAMP-DMB.

The crystal structure of DAMP-DMB reveals a dimeric association of mol­ecules around the inversion center, where the mol­ecules are connected through moderately strong N4—H4*B*⋯N2 [H⋯*A* = 2.19 (3) Å] hydrogen bonds (Fig. 2 ▸*a*, Table 1 ▸) (Steiner, 2002 ▸). In the dimeric association of DAMP-DMB mol­ecules, the ring pattern contains a total of eight atoms, two of them are donors, two are acceptors, hence the graph-set notation is 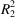 (8) (Bernstein *et al.*, 1995 ▸). These dimeric units are further stabilized by N—H⋯π inter­actions, specifically between the amine hydrogen atom of the pyrimidine ring and the π-electron cloud of the benzene ring [N3—H3*A*⋯*Cg*2, H⋯*Cg* = 2.89 (4) Å]. Similarly, as observed in the 2D finger print plots (see Section 4), the crystal structure also contains hydrogen-bonding inter­actions specifically, N—H⋯N inter­actions [N4—H4*A*⋯N1, H⋯*A* = 2.56 (3) Å]. Furthermore, the crystal structure exhibits inter­molecular H⋯H inter­actions involving the methyl hydrogen and and the hydrogen atom of the methyl­ene spacer. (Fig. 2 ▸*b*). This hierarchical organization, governed by multiple weak inter­molecular inter­actions, including H⋯H, N⋯H, C⋯H, and S⋯H, plays a crucial role in the overall packing and cohesion of the crystal structure.

## 4. Hirshfeld surface analysis

**Figure 3:**
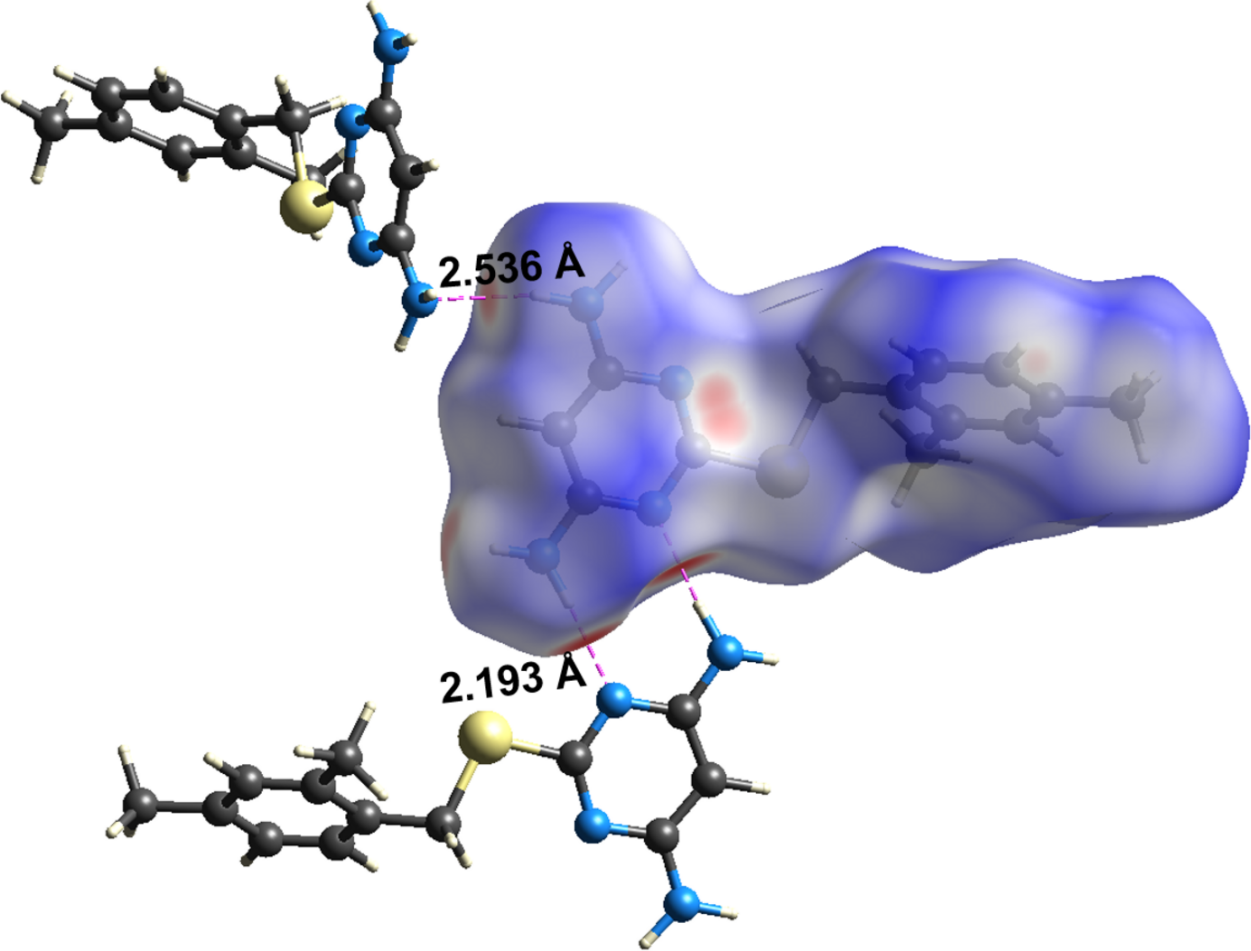
Visualization of the three-dimensional Hirshfeld surfaces for DAMP-DMB.

A Hirshfeld surface analysis (Spackman & Jayatilaka, 2009 ▸) was performed and fingerprint plots (Spackman & McKinnon, 2002 ▸) generated using *CrystalExplorer 21.5* (Spackman *et al.*, 2021 ▸) to investigate the inter­actions contributing to the cohesion of the crystal structure. The Hirshfeld surface and fingerprint plots are shown in Figs. 3 ▸ and 4 ▸. The presence of red spots on the Hirshfeld surface indicates close N⋯H contacts, which are also reflected in the corresponding 2D fingerprint plots. The mol­ecule predominantly engages in H⋯H, C⋯H, N⋯H, and S⋯H inter­actions, contributing 51.6%, 23.0%, 15.8%, and 8.5%, respectively to the Hirshfeld surface, accounting for 98.9% of the total inter­actions. In contrast, inter­actions such as C⋯C and C⋯N collectively account for only 0.9%, indicating their minimal role in crystal-structure cohesion. The 2D fingerprint plots reveals the presence of distinct hydrogen-bonding spikes corresponding to N—H⋯N inter­actions. The lower right spike at (*d*_i_, *d*_e_) = (1.2, 0.8), represents the hydrogen-bond acceptor, while the upper left spike at (*d*_i_, *d*_e_) = (0.8, 1.8) corresponds to the hydrogen-bond donor. Similarly, a sharp feature along the diagonal in the lower left region indicates a close H⋯H contact, shorter than 2.4 Å, where *d*_i_ = *d*_e_ ≃ 1.2 Å (Figs. 3 ▸ and 4 ▸).

## 5. Database survey

A survey of the Cambridge Structural Database (CSD, Version 5.45, last updated March 2024; Groom *et al.*, 2016 ▸) using ConQuest (Bruno *et al.*, 2002 ▸) revealed 32 crystal structures for the di­amino­pyrimidine-thiol (DAMP) fragment; among which, eleven structures are related to organometallic compounds. Out of the eleven structures, two complexes of the di­amino­pyrimidine thiol ligand with triphenyl tin and one with trimethyl tin are reported where the sulfur atom binds monodentately with the metal atom (CEHZIB, Grześkiewicz *et al.*, 2017 ▸; VUFTAT, VUFTEX, Ioannidou *et al.*, 2013 ▸). Similarly, three structures with ruthenium and two with cobalt metal centers are reported where the metal is coordinated bidentately with N and S atoms (FEGQER, Silva *et al.*, 2020 ▸; JACCAV, Ribeiro *et al.*, 2020 ▸; XOTDAO, da Silva *et al.*, 2019 ▸; TIYJUG01, Yamanari *et al.*, 2002 ▸; COHBEK, Gioftsidou *et al.*, 2024 ▸). Inter­estingly, one crystal structure with a Cu metal atom is reported where the di­amino­pyrimidine thiol derivative binds with the metal atom in a bidentate fashion through the nitro­gen atoms (DEDRAI, Moyaert *et al.*, 2017 ▸). Two structures of a di­amino­pyrimidine thiol derivative containing zinc are also deposited (TAGBUY, Romero *et al.*, 1990 ▸; ZIKFII, Salas *et al.*, 1995 ▸). Similarly, twelve crystal structures of DAMP with amides have been reported. In addition, one crystal structure having two DAMP fragments connected *via* a bridging methyl­ene (–CH_2_–) group are reported. There are also structures for methyl and ethyl derivatives directly connected to the thiol group of the DAMP fragment. However, no crystal structures of DAMP derivatives with 2,4-di­methyl­benzyl have been reported.

## 6. Synthesis and crystallization

A round-bottomed flask equipped with a magnetic stirrer was charged with di­amino­pyrimidine-thiol (50.0 mg, 0.351 mmol) dissolved in a mixture of 1.0 *N* aqueous NaOH (0.35 mL, 0.35 mmol) and methanol (5.0 mL). The reaction mixture was stirred at room temperature for 1 h and then concentrated *in vacuo* to afford a tan solid. The resulting solid was dissolved in DMF (5.0 mL), treated with 2,4-di­methyl­benzyl chloride (50.0 µL, 0.35 mmol), and stirred at room temperature for 2 h. The reaction progress was monitored by TLC. Upon completion, the DMF was removed *in vacuo*, and the residue was partitioned between water (50 mL) and chloro­form (3 × 50 mL). The combined organic extracts were dried over Na_2_SO_4_, filtered, and concentrated under vacuum. The residue was further dried at room temperature for 48 h, yielding the product as colorless crystals (90%) (Salieva *et al.*, 2025 ▸).

^1^H-NMR (600 MHz, CD_3_OD) δ: 2.23 (*s*, 3H, CH_3_), 2.30 (*s*, 3H, CH_3_), 4.27 (*s*, 2H, S-CH_2_) 5.29 (*s*, 1H, CH pyrimidine), 6.88 (*d*, *J* = 6 Hz, 1H, Ar), 6.93 (*s*, 1H, Ar), 7.17 (*d*, *J* = 12 Hz, H, CH Ar) ^13^C NMR (150 MHz, CD_3_OD) δ: 18.0, 19.7, 32.4, 79.2, 126.3, 129.7, 130.6, 132.3, 136.4, 136.7, 163.8, 169.6. LC-MC (Q-TOF) *m*/*z*; [*M*+*H*+] calculated C_13_H_17_N_4_S^+^ = 261.116, found 261.118.

Elemental analysis: calculated; C_13_H_16_N_4_S = 260.1168, C, 59.97; H, 6.19; N, 21.52; S, 12.31%. Found; C_13_H_16_N_4_S = 260.1168, C, 59.8882; H, 6.0750; N, 21.3749; S, 12.3001%.

## 7. Refinement

Crystal data, data collection and structure refinement details are summarized in Table 2 ▸. H atoms were refined isotropically by a mixture of independent and constrained refinement.

## Supplementary Material

Crystal structure: contains datablock(s) I. DOI: 10.1107/S2056989025002440/ex2091sup1.cif

Structure factors: contains datablock(s) I. DOI: 10.1107/S2056989025002440/ex2091Isup3.hkl

Supporting information file. DOI: 10.1107/S2056989025002440/ex2091Isup3.cml

CCDC reference: 2431935

Additional supporting information:  crystallographic information; 3D view; checkCIF report

## Figures and Tables

**Figure 4:**
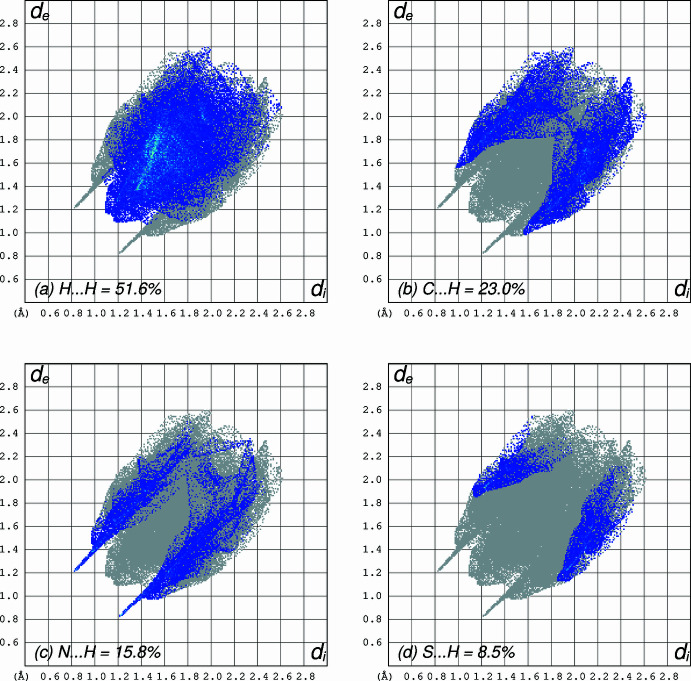
Two-dimensional fingerprint plots of the Hirshfeld surfaces for DAMP-DMB showing the contributions of various hydrogen-bonding inter­actions.

**Table 1:** Hydrogen-bond geometry (Å, °) *Cg*2 is the centroid of the C6–C11 ring.

*D*—H⋯*A*	*D*—H	H⋯*A*	*D*⋯*A*	*D*—H⋯*A*
C10—H10⋯C1^i^	0.93	2.82	3.623 (4)	145
N3—H3*B*⋯N4^ii^	0.82 (3)	2.54 (3)	3.340 (5)	168 (3)
N4—H4*A*⋯N1^iii^	0.87 (3)	2.56 (3)	3.372 (4)	156 (3)
N4—H4*A*⋯C4^iii^	0.87 (3)	2.70 (4)	3.540 (4)	164 (3)
N4—H4*B*⋯N2^iv^	0.86 (3)	2.19 (3)	3.039 (3)	172 (3)
N3—H3*A*⋯*Cg*2^v^	0.85 (4)	2.89 (4)	3.561 (3)	137 (3)

Symmetry codes: (i) 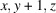; (ii) 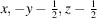; (iii) 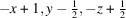; (iv) 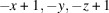; (v) 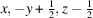.

**Table 2:** Experimental details

Crystal data	
Chemical formula	C_13_H_16_N_4_S
*M*_r_	260.36
Crystal system, space group	Monoclinic, *P*2_1_/*c*
Temperature (K)	293
*a*, *b*, *c* (Å)	14.482 (3), 9.3850 (19), 10.590 (2)
β (°)	108.07 (3)
*V* (Å^3^)	1368.3 (5)
*Z*	4
Radiation type	Cu *K*α
μ (mm^−1^)	2.00
Crystal size (mm)	0.2 × 0.1 × 0.07

Data collection	
Diffractometer	Bruker D8 VENTURE dual wavelength Mo/Cu
Absorption correction	Multi-scan (*SADABS*; Krause et al., 2015 ▸)
*T*_min_, *T*_max_	0.64, 0.87
No. of measured, independent and observed [*I* > 2σ(*I*)] reflections	37911, 2329, 2057
*R*_int_	0.040
(sin θ/λ)_max_ (Å^−1^)	0.595

Refinement	
*R*[*F*^2^ > 2σ(*F*^2^)], *wR*(*F*^2^), *S*	0.049, 0.149, 1.08
No. of reflections	2329
No. of parameters	181
H-atom treatment	H atoms treated by a mixture of independent and constrained refinement
Δρ_max_, Δρ_min_ (e Å^−3^)	0.57, −0.21

Computer programs: *APEX5* and *SAINT* (Bruker, 2016 ▸), *SHELXT2018/2* (Sheldrick, 2015*a* ▸), *SHELXL2018/3* (Sheldrick, 2015*b* ▸) and *OLEX2* (Dolomanov *et al.*, 2009 ▸).

## supplementary crystallographic information

### 2-[(2,4-Dimethylbenzyl)sulfanyl]pyrimidine-4,6-diamine Crystal data

**Table:**

C_13_H_16_N_4_S	*F*(000) = 552
*M**_r_* = 260.36	*D*_x_ = 1.264 Mg m^−^^3^
Monoclinic, *P*2_1_/*c*	Cu *K*α radiation, λ = 1.54178 Å
*a* = 14.482 (3) Å	Cell parameters from 9926 reflections
*b* = 9.3850 (19) Å	θ = 5.7–66.3°
*c* = 10.590 (2) Å	µ = 2.00 mm^−^^1^
β = 108.07 (3)°	*T* = 293 K
*V* = 1368.3 (5) Å^3^	Prism, colourless
*Z* = 4	0.2 × 0.1 × 0.07 mm

### 2-[(2,4-Dimethylbenzyl)sulfanyl]pyrimidine-4,6-diamine Data collection

**Table:**

Bruker D8 VENTURE dual wavelength Mo/Cu diffractometer	2329 independent reflections
Radiation source: microfocus sealed X-ray tube, INCOATEC IµS	2057 reflections with *I* > 2σ(*I*)
Graphite monochromator	*R*_int_ = 0.040
Detector resolution: 7.3910 pixels mm^-1^	θ_max_ = 66.6°, θ_min_ = 5.7°
φ and ω scans	*h* = −16→16
Absorption correction: multi-scan (SADABS; Krause et al., 2015)	*k* = −11→11
*T*_min_ = 0.64, *T*_max_ = 0.87	*l* = −12→12
37911 measured reflections	

### 2-[(2,4-Dimethylbenzyl)sulfanyl]pyrimidine-4,6-diamine Refinement

**Table:**

Refinement on *F*^2^	Secondary atom site location: difference Fourier map
Least-squares matrix: full	Hydrogen site location: mixed
*R*[*F*^2^ > 2σ(*F*^2^)] = 0.049	H atoms treated by a mixture of independent and constrained refinement
*wR*(*F*^2^) = 0.149	*w* = 1/[σ^2^(*F*_o_^2^) + (0.068*P*)^2^ + 0.8542*P*] where *P* = (*F*_o_^2^ + 2*F*_c_^2^)/3
*S* = 1.08	(Δ/σ)_max_ < 0.001
2329 reflections	Δρ_max_ = 0.57 e Å^−^^3^
181 parameters	Δρ_min_ = −0.21 e Å^−^^3^
0 restraints	

### 2-[(2,4-Dimethylbenzyl)sulfanyl]pyrimidine-4,6-diamine Special details

**Table:**

Geometry. All esds (except the esd in the dihedral angle between two l.s. planes) are estimated using the full covariance matrix. The cell esds are taken into account individually in the estimation of esds in distances, angles and torsion angles; correlations between esds in cell parameters are only used when they are defined by crystal symmetry. An approximate (isotropic) treatment of cell esds is used for estimating esds involving l.s. planes.

### 2-[(2,4-Dimethylbenzyl)sulfanyl]pyrimidine-4,6-diamine Fractional atomic coordinates and isotropic or equivalent isotropic displacement parameters (Å^2^)

**Table:**

	*x*	*y*	*z*	*U*_iso_*/*U*_eq_	
S1	0.70925 (6)	0.22323 (8)	0.45344 (7)	0.0692 (3)	
N2	0.58768 (15)	0.0155 (2)	0.4032 (2)	0.0564 (5)	
N1	0.66759 (16)	0.0540 (2)	0.2395 (2)	0.0577 (5)	
N3	0.6348 (2)	−0.0867 (4)	0.0539 (3)	0.0772 (8)	
N4	0.4784 (2)	−0.1665 (3)	0.3859 (3)	0.0711 (7)	
C4	0.64938 (18)	0.0803 (3)	0.3523 (2)	0.0531 (6)	
C1	0.61661 (19)	−0.0562 (3)	0.1686 (2)	0.0562 (6)	
C2	0.5520 (2)	−0.1347 (3)	0.2127 (3)	0.0598 (7)	
H2	0.518848	−0.211765	0.164224	0.072*	
C3	0.53829 (19)	−0.0950 (3)	0.3309 (2)	0.0555 (6)	
C6	0.81963 (19)	0.4397 (3)	0.4079 (3)	0.0598 (7)	
C7	0.88069 (19)	0.4817 (3)	0.5314 (3)	0.0623 (7)	
C8	0.8975 (2)	0.6268 (4)	0.5536 (3)	0.0770 (8)	
H8	0.937665	0.657394	0.635863	0.092*	
C9	0.8560 (3)	0.7274 (3)	0.4561 (4)	0.0847 (10)	
C10	0.7976 (2)	0.6828 (4)	0.3357 (4)	0.0923 (11)	
H10	0.770351	0.749011	0.269275	0.111*	
C11	0.7789 (2)	0.5421 (4)	0.3123 (4)	0.0805 (9)	
H11	0.737651	0.513470	0.230042	0.097*	
C5	0.7966 (2)	0.2853 (3)	0.3734 (3)	0.0692 (8)	
H5A	0.855552	0.228946	0.403493	0.083*	
H5B	0.769744	0.274625	0.277885	0.083*	
C12	0.9274 (3)	0.3764 (4)	0.6386 (3)	0.0899 (10)	
H12A	0.980309	0.421258	0.704739	0.135*	
H12B	0.951482	0.297020	0.600929	0.135*	
H12C	0.880392	0.343347	0.678883	0.135*	
C13	0.8798 (4)	0.8847 (4)	0.4839 (6)	0.1331 (18)	
H13A	0.849016	0.939278	0.405419	0.200*	
H13B	0.948811	0.898122	0.508998	0.200*	
H13C	0.856385	0.915894	0.554811	0.200*	
H3A	0.675 (3)	−0.036 (4)	0.030 (3)	0.085 (11)*	
H3B	0.603 (2)	−0.150 (4)	0.007 (3)	0.070 (10)*	
H4A	0.437 (2)	−0.222 (3)	0.332 (3)	0.071 (9)*	
H4B	0.466 (2)	−0.124 (3)	0.450 (3)	0.072 (9)*	

### 2-[(2,4-Dimethylbenzyl)sulfanyl]pyrimidine-4,6-diamine Atomic displacement parameters (Å^2^)

**Table:**

	*U*^11^	*U*^22^	*U*^33^	*U*^12^	*U*^13^	*U*^23^
S1	0.0831 (5)	0.0680 (5)	0.0734 (5)	−0.0215 (3)	0.0488 (4)	−0.0164 (3)
N2	0.0670 (13)	0.0534 (12)	0.0586 (12)	−0.0058 (10)	0.0338 (10)	−0.0031 (10)
N1	0.0677 (13)	0.0592 (12)	0.0537 (12)	−0.0022 (10)	0.0296 (10)	0.0014 (10)
N3	0.096 (2)	0.0867 (19)	0.0632 (15)	−0.0224 (16)	0.0454 (15)	−0.0157 (14)
N4	0.0889 (18)	0.0677 (15)	0.0719 (16)	−0.0251 (14)	0.0468 (14)	−0.0155 (13)
C4	0.0594 (14)	0.0509 (13)	0.0554 (14)	0.0026 (11)	0.0274 (11)	0.0025 (11)
C1	0.0633 (15)	0.0597 (15)	0.0500 (13)	0.0048 (12)	0.0240 (11)	0.0012 (11)
C2	0.0680 (16)	0.0579 (15)	0.0597 (15)	−0.0052 (12)	0.0288 (12)	−0.0069 (12)
C3	0.0626 (15)	0.0524 (14)	0.0581 (14)	0.0009 (11)	0.0286 (12)	0.0027 (11)
C6	0.0559 (15)	0.0639 (16)	0.0697 (17)	−0.0036 (12)	0.0345 (13)	0.0030 (13)
C7	0.0552 (15)	0.0678 (17)	0.0722 (17)	−0.0032 (12)	0.0317 (13)	0.0024 (13)
C8	0.0640 (18)	0.083 (2)	0.089 (2)	−0.0123 (15)	0.0304 (15)	−0.0116 (17)
C9	0.077 (2)	0.0590 (18)	0.129 (3)	0.0018 (15)	0.047 (2)	0.0057 (19)
C10	0.067 (2)	0.086 (2)	0.119 (3)	0.0026 (17)	0.023 (2)	0.031 (2)
C11	0.0671 (18)	0.085 (2)	0.089 (2)	−0.0080 (16)	0.0251 (16)	0.0186 (18)
C5	0.0748 (18)	0.0703 (18)	0.0786 (19)	−0.0111 (14)	0.0473 (15)	−0.0064 (14)
C12	0.080 (2)	0.111 (3)	0.078 (2)	−0.0021 (19)	0.0240 (17)	0.020 (2)
C13	0.129 (4)	0.072 (2)	0.197 (5)	−0.006 (2)	0.048 (4)	−0.008 (3)

### 2-[(2,4-Dimethylbenzyl)sulfanyl]pyrimidine-4,6-diamine Geometric parameters (Å, º)

**Table:**

S1—C4	1.767 (3)	C7—C8	1.390 (4)
S1—C5	1.823 (3)	C7—C12	1.500 (4)
N2—C4	1.326 (3)	C8—H8	0.9300
N2—C3	1.354 (3)	C8—C9	1.390 (5)
N1—C4	1.323 (3)	C9—C10	1.359 (5)
N1—C1	1.354 (3)	C9—C13	1.524 (5)
N3—C1	1.351 (3)	C10—H10	0.9300
N3—H3A	0.85 (4)	C10—C11	1.355 (5)
N3—H3B	0.82 (3)	C11—H11	0.9300
N4—C3	1.362 (3)	C5—H5A	0.9700
N4—H4A	0.87 (3)	C5—H5B	0.9700
N4—H4B	0.86 (3)	C12—H12A	0.9600
C1—C2	1.381 (4)	C12—H12B	0.9600
C2—H2	0.9300	C12—H12C	0.9600
C2—C3	1.379 (4)	C13—H13A	0.9600
C6—C7	1.390 (4)	C13—H13B	0.9600
C6—C11	1.388 (4)	C13—H13C	0.9600
C6—C5	1.505 (4)		
			
C4—S1—C5	103.99 (13)	C9—C8—H8	119.1
C4—N2—C3	115.2 (2)	C8—C9—C13	119.7 (4)
C4—N1—C1	114.6 (2)	C10—C9—C8	119.2 (3)
C1—N3—H3A	119 (2)	C10—C9—C13	121.1 (4)
C1—N3—H3B	118 (2)	C9—C10—H10	119.9
H3A—N3—H3B	122 (3)	C11—C10—C9	120.2 (3)
C3—N4—H4A	115 (2)	C11—C10—H10	119.9
C3—N4—H4B	115 (2)	C6—C11—H11	119.1
H4A—N4—H4B	122 (3)	C10—C11—C6	121.7 (3)
N2—C4—S1	111.56 (18)	C10—C11—H11	119.1
N1—C4—S1	119.41 (19)	S1—C5—H5A	109.8
N1—C4—N2	129.0 (2)	S1—C5—H5B	109.8
N1—C1—C2	122.0 (2)	C6—C5—S1	109.16 (19)
N3—C1—N1	115.8 (3)	C6—C5—H5A	109.8
N3—C1—C2	122.1 (3)	C6—C5—H5B	109.8
C1—C2—H2	121.1	H5A—C5—H5B	108.3
C3—C2—C1	117.8 (2)	C7—C12—H12A	109.5
C3—C2—H2	121.1	C7—C12—H12B	109.5
N2—C3—N4	115.5 (2)	C7—C12—H12C	109.5
N2—C3—C2	121.4 (2)	H12A—C12—H12B	109.5
N4—C3—C2	123.0 (3)	H12A—C12—H12C	109.5
C7—C6—C5	122.0 (3)	H12B—C12—H12C	109.5
C11—C6—C7	119.5 (3)	C9—C13—H13A	109.5
C11—C6—C5	118.5 (3)	C9—C13—H13B	109.5
C6—C7—C8	117.7 (3)	C9—C13—H13C	109.5
C6—C7—C12	122.1 (3)	H13A—C13—H13B	109.5
C8—C7—C12	120.2 (3)	H13A—C13—H13C	109.5
C7—C8—H8	119.1	H13B—C13—H13C	109.5
C7—C8—C9	121.8 (3)		
			
N1—C1—C2—C3	1.8 (4)	C7—C6—C5—S1	76.3 (3)
N3—C1—C2—C3	−179.9 (3)	C7—C8—C9—C10	−0.2 (5)
C4—S1—C5—C6	154.6 (2)	C7—C8—C9—C13	−177.8 (3)
C4—N2—C3—N4	176.9 (2)	C8—C9—C10—C11	1.1 (5)
C4—N2—C3—C2	−0.4 (4)	C9—C10—C11—C6	−1.3 (5)
C4—N1—C1—N3	−179.1 (3)	C11—C6—C7—C8	0.4 (4)
C4—N1—C1—C2	−0.8 (4)	C11—C6—C7—C12	−179.7 (3)
C1—N1—C4—S1	−178.90 (18)	C11—C6—C5—S1	−104.2 (3)
C1—N1—C4—N2	−1.1 (4)	C5—S1—C4—N2	175.2 (2)
C1—C2—C3—N2	−1.2 (4)	C5—S1—C4—N1	−6.7 (3)
C1—C2—C3—N4	−178.3 (3)	C5—C6—C7—C8	179.9 (2)
C3—N2—C4—S1	179.62 (18)	C5—C6—C7—C12	−0.2 (4)
C3—N2—C4—N1	1.7 (4)	C5—C6—C11—C10	−179.0 (3)
C6—C7—C8—C9	−0.6 (4)	C12—C7—C8—C9	179.5 (3)
C7—C6—C11—C10	0.5 (5)	C13—C9—C10—C11	178.8 (4)

### 2-[(2,4-Dimethylbenzyl)sulfanyl]pyrimidine-4,6-diamine Hydrogen-bond geometry (Å, º)

*Cg*2 is the centroid of the C6–C11 ring.

**Table:**

*D*—H···*A*	*D*—H	H···*A*	*D*···*A*	*D*—H···*A*
C10—H10···C1^i^	0.93	2.82	3.623 (4)	145
N3—H3*B*···N4^ii^	0.82 (3)	2.54 (3)	3.340 (5)	168 (3)
N4—H4*A*···N1^iii^	0.87 (3)	2.56 (3)	3.372 (4)	156 (3)
N4—H4*A*···C4^iii^	0.87 (3)	2.70 (4)	3.540 (4)	164 (3)
N4—H4*B*···N2^iv^	0.86 (3)	2.19 (3)	3.039 (3)	172 (3)
N3—H3*A*···*Cg*2^v^	0.85 (4)	2.89 (4)	3.561 (3)	137 (3)

Symmetry codes: (i) *x*, *y*+1, *z*; (ii) *x*, −*y*−1/2, *z*−1/2; (iii) −*x*+1, *y*−1/2, −*z*+1/2; (iv) −*x*+1, −*y*, −*z*+1; (v) *x*, −*y*+1/2, *z*−1/2.
